# Prostate Cancer-based Interventions’ Efficacy on Knowledge and Adherence Intention to Healthy Lifestyle among Men

**DOI:** 10.31557/APJCP.2020.21.4.1129

**Published:** 2020-04

**Authors:** Ahmad M Saleh, Wasileh Petro-Nustas, Elturabi Elsayed Ebrahim, Arul Vellaiyan, Yahya Najjar, Mariam Awad Mazyad Almutairi

**Affiliations:** 1 *Prince Sattam Bin Abdulaziz University, College of Applied Medical Sciences, Department of Nursing, Alkharj, Riyadh, Saudi Arabia, *; 2 *University of Texas, Faculty of Nursing, Arlington, USA, *; 3 *Al-Balqa Applied University, Faculty of Nursing, Amman, Jordan. *

**Keywords:** Prostate Cancer, knowledge, adherence intention, healthy lifestyle, nursing intervention

## Abstract

**Background:**

Previous Literature has supported educational program efficacy, but no studies have been found to examine Prostate Cancer-based interventions’ Efficacy on knowledge and adherence intention to a healthy lifestyle among Men in Jordan.

**Purpose:**

The purpose of the current study was to assess Prostate Cancer-based interventions’ efficacy on knowledge and adherence intention to a healthy lifestyle among Men in Jordan.

**Methods:**

A quasi-experimental research with one group pretest-posttest design was used and the study was conducted in Masjids (praying place), Amman, Jordan. The population consists of Jordanian Men aged 40 years and above. The sample size was 76 men, who fulfilled the inclusion criteria. The tool used for data collection was a structured questionnaire.

**Results:**

Paired sample t-test showed that the change in the mean knowledge scores (9.5), p < .001 was statistically significant 1 month after the application of the program. In addition, the change in the mean adherence to healthy lifestyle scores (4.7), p < .05 was statistically significant 1 month after the application of the structured teaching program.

**Conclusion:**

Jordanian men had gained knowledge and Adherence Intention to Healthy Lifestyle regarding prostate cancer after the implementation of a structured teaching program.

**Implications for Practice:**

Educational program aimed at motivating men to increase their knowledge of prostate cancer and having adherence intention to a healthy lifestyle.

## Introduction

Cancer is a major health problem, being the second reason of death in developing nations (Nair, 2018). Cancers’ burden is increasing in developing societies as a consequence of rapid population growth, poverty, the prevalence of cancer-associated infections, and cancer-associated lifestyle choices, including higher tobacco consumption, lack of activity, and westernized diets (Smith et al., 2018). Accurate statistics on cancer incidence and outcomes are essential, not only to identify causes, but also to develop, implement, and evaluate cancer prevention programs (Ferlay et al., 2010). It is also significant to consider an evaluation tool of the future national financial burden caused by rising cancer incidence; the estimated annual cancer deaths are expected to increase from 7.6 million to the 13 million, from the year 2008 to the year 2030 (World Health Organization, 2012). In Jordan, a national population-based cancer registry began in 1996 with the founding of the Jordan Cancer Registry (JCR) under the supervision of the Ministry of Health. Cancer incidence in Jordan has increased to 46% in the past decade, from 3,362 cases in 2000 to 4,921 in 2010 (Tarawneh et al., 2010). Overall, 32.4% of new cancer cases in 2010 were diagnosed among elderly individuals (≥65 years) who account for 3.3% of the Jordanian population. Distribution of new cancer cases by age group revealed that 4% of the cases were those under 15 years, 25% were 15–44 years, and 38.4% were 45–64 years (Tarawneh et al., 2010).

Globally, Prostate Cancer (PC) considered the sixth leading reason of cancer death in males, and the eleventh leading reason of death from cancer in all groups age (Torre et al., 2015; Nair, 2018). In Jordan, PC is the third most frequently occurring cancer among males, with 218 new cases in 2010 (9.4%) and the third most common cause of death in males about (6.2%) of total deaths in 2010 (Tarawneh et al., 2010).

The American Cancer Society (ACS) advises receiving annual digital rectal exams (DREs) and prostate-specific antigen (PSA) tests, starting at age 45 years for at-risk groups comprising individuals with first degree relatives diagnosed with PC at an early age. Others should be screened annually from 50 years onwards (ACS, 2018b). Risk factors for PC include being male, aging, family history (Hx), high-fat product consumption, genetic changes, and obesity. Other potential risk factors such as smoking, being a firefighter, prostatitis, sexually transmitted infections (STI), and vasectomy did not demonstrate as clear relation to PC (ACS, 2018a). Modification of risk factors, adherence intention to a healthy lifestyle, and early screening contribute to reducing PC progression (NCI, 2012; ACS, 2018a).

Therefore, patients need accessible and adequate health education regarding these preventive and diagnostic strategies. Studies on patient education designed to raise knowledge and adherence intention to a healthy lifestyle among men show that brief and print-based interventions enhance knowledge of symptoms and risk factors (Moyer, 2012), as well as rates of screening (Taylor et al., 2006; Taylor et al., 2016). No studies yet have addressed prostate-related educational program conducted in Jordan. Therefore, it is essential to examine the PC educational program impact on knowledge, and adherence intention to a healthy lifestyle among Jordanian men.

*Literature review*

Globally, prostate cancer is the second most frequently diagnosed cancer among males (899,000 cases or 13.6% of male cancers recorded). Nearly three-quarters of registered cases— 644,000 cases—are from developing countries (Nair, 2018). PC incidence rates vary by region and continent, largely due to distinctions in the presence of PCS and subsequent biopsy facilities that are more widespread in Europe and North America (Nair, 2018). Yet, the incidence rates are relatively high in certain developing regions, for example, the Caribbean, Sub Africa, and South America (Nair, 2018). With an estimated 258,000 deaths worldwide in the year 2008; which is (6.1%) of all cancers (Nair, 2018).

Many studies have evaluated the efficacy of PC educational resources based on the level of knowledge, and adherence intention to a healthy lifestyle (Drake al., 2010; Ukoli et al., 2013; Keane, 2015). Drake et al., (2010) employed a quasi-experimental design to examine the impact of the small group education session on the level of knowledge about PC screening and self-efficacy, so as to engage in the Informed Decision-Making (IDM) process. 73 participants were joined and the intervention was given by an AA health educator. The intervention included a tool named the ‘Road Map’ that depicts the potential sequels of a decision to undergo screening. The intervention was guided by the Ottawa Decision Support Framework and the HBM. 

The findings showed that PC knowledge (p < 0.0001) and self-efficacy (p = 0.025) significantly increased. The study concluded that a church-based intervention delivered by an AA health educator is a hopeful strategy for promoting IDM among AA men. Additionally, Sheehan (2009) examined the efficacy of a brief digital video intervention on the level of knowledge and the perceived individual risk of evolving PC among 123 Caucasian men, aged 45–75 years. Men recorded significantly higher on a knowledge scale and were significant, more able to exactly rate their individual risk after watching the video. Furthermore, a significantly greater number of men intended to discuss PCS with their health care provider post-intervention. Moreover, this study demonstrated that nursing interventions aimed at educating men about PC and PCS which do not need to be time-consuming to be effective. Moreover, Husaini et al., (2008) employed a quasi-experimental delayed-control (crossover) design with randomization at the church level. 45 AA churches were randomly divided into two study groups: early intervention and delayed intervention. A convenience sample of 430 AA male volunteers (ages 40–70) was enrolled through the churches. The intervention was a culturally tailored group’s educational program, which included a video and a question-and-answer session with an AA physician. Inside each group, the level of knowledge, screening prevalence, and perceived threat increased significantly. However, the findings propose that the delayed-intervention group did not operate as a pure control group, and may have accidentally received a partial intervention.

Additionally, Taylor et al., (2006) carried out a randomized intervention study of 238 AA men, aged between 40–70 years, to determine the effectiveness of the educational interventions, a printed booklet, and videotape relative to waitlist control, on the knowledge and decisions conflict, regarding PCS. Intervention materials were mailed to men at home. Assessments were taken at baseline, 1 month, and 12 months after intervention. The results indicated that the video and booklet significantly improved the level of knowledge and lessened decisional conflict in the experimental group, compared to the control waitlist group. In addition, Ruthman and Ferrans (2004) conducted a quasi-experimental study to test the effectiveness of a video in teaching patients about PCS and treatment in a clinical setting. Knowledge level increased significantly from pretest to posttest for the experimental group (p < 0.001), but not for the control group. 

More patients in the experimental group modified their preference for PSA testing (31% experimental vs. 2% control, p = 0.002), indicating that patients were influenced by the information presented. Likewise, a study implemented by Wilkinson et al., (2003) to identify whether an educational program on the PC would affect the level of knowledge and awareness among 835 AA men, the results revealed a significant improvement in the level of knowledge post-seminar scores. Since knowledge levels among AA men were initially low, Wilkinson and colleagues concluded that a culturally relevant educational program could dramatically improve the level of knowledge and awareness of PC.

In Jordan, to the best of the researchers’ knowledge, there are no research studies being conducted to assess prostate cancer-based interventions’ efficacy on knowledge and adherence intention to a healthy lifestyle among Men in Jordan. Therefore, increasing PC knowledge, beliefs, and intention, among Jordanian men and providing men with appropriate information to enable them to make an informed decision, continues to be an important part in improving Prostate cancer screening in this group.

## Materials and Methods

Prostate Cancer-based interventions’ Efficacy on knowledge and Adherence Intention to Healthy Lifestyle among Men in Jordan was examined using quasi-experimental research with one group pretest-posttest design.

*Sample and Setting*

A convenience sampling technique was used to recruit the participants, who visited Masjids (praying place). The inclusion criteria for participation in the study included (a) men aged 40 years and above (PCC, 2015; ACS, 2018b) who live in Amman; (b) able to read, hear, understand, and speak the Arabic language. Men with a previous diagnosis of PC are excluded from the study, because of possible confounding knowledge of the disease, thus, it is considered as the only exclusion criterion. The sample size was calculated by using G* power 3.0 software (Faul et al., 2007). Using a power level of 0.80, an alpha level of 0.05, and an effect size of 0.30 for a two-tailed paired t-test, the estimated sample size was 70. With an estimated attrition rate of 25% (Polit and Beck, 2010), an additional 18 participants were included for a total sample size of 88 study participants.

*Data Collection and Procedure*

The study method and protocol were reviewed and approved by the ethical committee in the faculty of nursing at the University of Jordan, and the Ministry of Islamic Awqaf Trust Affairs.

Written informed consent was obtained from all participants who agreed to participate in the study. All participants were reviewed by the primary researcher to ensure the eligibility of the participants to participate in the study. After that, the written informed consent was obtained from each participant. Then, the primary researcher collected the data concerning the knowledge, and adherence intention to a healthy lifestyle at zero weeks, these data were collected from 88 men. After that, the primary researcher implemented the prostate cancer educational program immediately. One month after the program application, the primary researcher collected the posttest data from 76 participants.

*Instrumentation*

A structured questionnaire was utilized for collecting the data to achieve the purpose of the study. The questionnaire started with a brief statement concerning the purpose of the study, informed consent, and followed by three parts. Part one is the demographic, which consists of a checklist multiple choice, and gap filling questions type concerning all variables like age, gender, monthly income, and educational level. The second part is The Knowledge of Prostate Cancer and the adherence intention to healthy lifestyle scale.

*The knowledge of the PC screening questionnaire*

A translated version of the knowledge of the PC screening questionnaire developed by Weinrich et al., (2004), was used to measure participants’ knowledge about PC and PCS. 12 items were used to measure knowledge about PCS limitations, PC symptoms, PC risk factors; side effect from treatment and screening age guidelines. 

An overall knowledge score was computed by totaling the number of correct responses, with a possible range from 0 to 12, and higher scores indicating greater knowledge. Items were tested for internal consistency reliability in the current study and the results revealed that Cronbach’s α coefficient was 0.77, prior to PC educational program, while it was 0.81 for the total scale, post PC educational program.

*Adherence Intention to Healthy Lifestyle scale*

The translated scale was developed based on the guidelines given by Francis et al., (2004), to measure the generalized intention regarding PCS. The PCS intention scale is composed of three items indicating adherence intention to healthy lifestyle, presented in the Arabic language and measured on a five-point Likert scale (“strongly disagree = 1” to “strongly agree = 5”) with total scores ranging from 3–15, with higher scores indicating higher degree of adherence intention to healthy lifestyle. Items were tested for internal consistency reliability by Cronbach’s α coefficient which was reported in previous studies to be around 0.95. Items were tested for internal consistency reliability in the current study and the results revealed that Cronbach’s α coefficient was 0.95 for the total scale, prior to PC educational program, while it was 0.83 for the total scale, post prostate educational program. In addition, content validity was tested in the previous study based on a matrix suggested by Saleh et al., (2015).

The Permission to use the original and translated questionnaires was obtained from the authors, the translated versions were reviewed by another a group of Jordanian faculty members for proper language use and cultural appropriateness. The questionnaires were pilot tested with 20 participants who met the inclusion criteria of the study. 

The estimated time to complete the questionnaires was 20-30 min. Face validity of the two instruments was assessed by four experts in the area of Prostate Cancer. The results revealed that the two instruments were valid and measuring what was supposed to measure.

*The Prostate Educational Program*

The prostate cancer educational program took approximately 1-hour educational session consisting of a 30-minute lecture that was conducted by the researcher, a booklet and brochure, that summarized the material provided by an investigator and a 30-minute interactive group discussion. Some individualized sensitive questions were answered individually. The brochure “Prostate Cancer: What you should know about prostate cancer” was adopted from Medical Cancer Center. This educational module was written in the Arabic language and was derived from relevant literature in Evidence-Based Practice (ACS, 2018a).

The brochure was developed and reviewed by a multidisciplinary team of an oncologist, oncology nurse educator, clinical nurse specialist, laboratory technician, and radiologist. Booklet educational material was developed by the researcher to complement the information missed in the brochure. Both booklet and brochure materials were evaluated by a panel of experts, including two urologists, consultant clinical oncology, and one nurse, with over 7 years’ experience in oncology critical care, to ensure the adequacy of the information that was provided to the participants. 

The educational booklet and the brochure covered the information related to the overview of the prostate gland, an overview of neoplasm, risk factors for developing PC, PCS, signs, and symptoms of PC, diagnosis, grading scale for diagnosing PC, treatments and its side effects, follow up caring and preventive measures.

*Data Analysis*

The Statistical Package for the Social Science (SPSS) software, version 21 was used to analyze the study data (International Business Machines Corporation, 2012). Descriptive statistics were used to describe the sample characteristics. Independent sample t-test was used to assess whether or not there were statistically significant differences in the level of knowledge, and adherence intention to healthy lifestyle scores between experimental and control group after the implementation of the prostate cancer educational program.

## Results

*Sample Characteristics*

**Figure 1:**
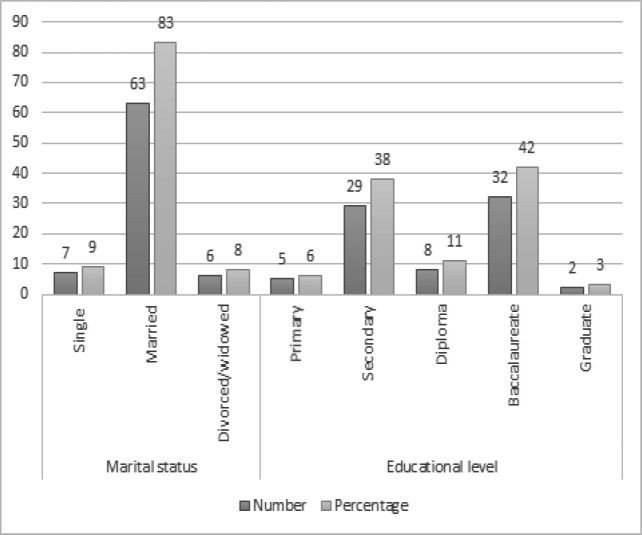
Sample Characteristics; Number and Percentage of Participants (N= 76).

One hundred and fifty-four participants were involved in the study analysis, indicating a response rate of 74%. The mean age of participants was 53.1 years (SD = 9.52) and ranged between 40-years old and 78-years old years. Most of the respondents (83%) were married, followed by (9%) who were single and (8%) who were divorced or widowed. In addition, (42%) had a baccalaureate level of education, (38%) had a secondary educational level, (6%) had a primary educational level, (3%) had a graduate educational level, and (11%) had a diploma level of education. The sample characteristics are presented in Figure 1. Concerning those participants who did not complete the entire study (N = 12) were like those participants who completed the entire study. 

*Level of Knowledge and Adherence Intention to Healthy Lifestyle*

A paired sample t-test was conducted to evaluate the mean scores of participants knowledge and adherence intention to a healthy lifestyle on two different occasions (baseline and one month later). The results revealed that the participants gained knowledge regarding PC. Participants showed significant increases in knowledge scores from the baseline phase-1 (M = 5.08, SD = 2.988) to the 1-month follow-up phase-2 [M = 9.5, SD = 2.63, t (75) = -23.966, p < 0.001]. The effect size as indexed by η2, was 0.88, a large effect size (Warner, 2008). 

In addition, results showed a significant increase in adherence intention to healthy lifestyle scores regarding PC from the baseline phase-1 (M = 3.55, SD = 0.87) to the 1-month follow-up phase- 2 [M = 4.7, SD = 1.77, t (75) = -2.32, p < 0.05]. The effect size as indexed by η^2^ was 0.67; large effect size (Warner, 2008). Findings of the PC educational program efficacy on the levels of knowledge and adherence intention to a healthy lifestyle are presented in Table 1. The change in the mean scores between the pretest and the posttest are reported in this Table.

**Table 1:** A Paired T-test on the Level of Knowledge and Adherence Intention to Healthy Lifestyle Acquisition among Participants

Variable	Mean change (SD)	t	*P*-value
Pretest knowledge	5.08 (2.99)	-23.97	0.001
Posttest Knowledge	8.7 (2.422)		
Pretest adherence intention to healthy lifestyle	3.55 (0.87)	-2.32	0.023
Posttest adherence intention to a healthy lifestyle	3.71 (0.77)		

Note, SD, Standard Deviation

## Discussion

The results in the current study support educational program efficacy in gaining knowledge among Jordanian men in Amman at one month after the implementation of the educational program. This finding is consistent with the findings of many studies (Drake et al., 2010; Keane, 2015; Ivlev et al., 2018). which showed that the educational program significantly improved cancer-related knowledge. The significant effect of the cancer prostate educational program on knowledge may be attributed to many factors. The systematic education which included a combination of verbal information, booklet and brochure help improve patients’ knowledge. The educational program language and content is appropriate to the individual in terms of gender, age, Jordanian culture, and other socioeconomic factors. The previous factors have an important impact on the ability of individuals to learn (Drake et al., 2010). In addition, the using of individualized discussion after the application of the educational program and giving written information may have contributed to the success of the intervention. 

Regarding the effect of educational program efficacy on adherence intention to a healthy lifestyle, the results showed that the adherence intention to healthy lifestyle variable improved significantly after 1 month form the application of the educational program. This finding is congruent with the finding of the previous research studies (Odedina et al., 2014; Keane, 2015; Ivlev et al., 2018), which showed that educational program significantly improved the adherence intention to a healthy lifestyle. An important possible explanation for the improvement in the adherence intention to a healthy lifestyle in the present study is the significant improvement in the level of knowledge regarding prostate cancer after the application of the educational program. This is congruent with the findings of many studies (Mahmoud, and Elderiny, 2018; Klusmann, and Notthoff, 2018) that showed a positive correlation between knowledge and adherence intention to a healthy lifestyle. Improved knowledge level associated with illness and treatments has been revealed to enhance adherence intention to a healthy lifestyle (Alikari et al., 2019).

*Limitations*

Convenience sampling as well as limiting the study in Amman posed a problem for the generalizability of the findings to all Jordanian men. Short follow-up period to measure the concept of knowledge and adherence intention to healthy lifestyle were another limitation. adherence intention to a healthy lifestyle is needed to be measured over a long period of time. The use of self-reported behavior tool were other limitations of the present study.

*Conclusion and Recommendations*

The application of this study in practice may help improve the knowledge and the adherence intention to a healthy lifestyle among Jordanian men in Amman. Conducting research exploring educational program efficacy on the level of knowledge and adherence intention to a healthy lifestyle for prostate cancer may provide a basis for conducting other studies which can address the gap and the limitations of the present study. The findings from this study deserve to be replicated using a larger and more heterogeneous randomly selected sample.

Prostate cancer educational program efficacy on knowledge and adherence intention to healthy lifestyle were still of reasonable magnitude 1 month after the program application. Further research is necessary to measure long-term time after the application of such this educational program. Findings of this study confirm the importance of the prostate educational program in improving the knowledge and adherence intention to a healthy lifestyle.

## References

[B1] Alikari V, Tsironi M, Matziou V (2019). The impact of education on knowledge, adherence and quality of life among patients on haemodialysis. Qual Life Res.

[B2] American Cancer Society (ACS) (2018a) Prostate Cancer Overview.

[B3] American Cancer Society (ACS) (2018b) Prostate Cancer Prevention and Early Detection.

[B4] Drake BF, Shelton RC, Gilligan T, Allen SJ (2010). A church-based intervention to promote informed decision making for prostate cancer screening among African American men. J Natl Med Assoc.

[B5] Faul F, Erdfelder E, Lang AG, Buchner A (2007). G* Power 3: A flexible statistical power analysis program for the social, behavioral, and biomedical sciences. Behav Res Methods.

[B6] Ferlay J, Shin HR, Bray F (2010). Estimates of worldwide burden of cancer in 2008: GLOBOCAN 2008. Int J Cancer.

[B7] Francis J, Eccles MP, Johnston M (2004). Constructing questionnaires based on the theory of planned behaviour: A manual for health services researchers. Centre for Health Services Research.

[B8] Husaini BA, Reece MC (2008). A church-based program on prostate cancer screening for African American men: Reducing Health Disparities. Mortality.

[B9] Husaini BA, Reece MC Emerson JS (2008). A church-based program on prostate cancer screening for African American men: reducing health disparities. Ethn Dis.

[B11] Ivlev I, Jerabkova S, Mishra M, Cook LA, Eden KB 2018) Prostate cancer screening patient decision aids: a systematic review and meta-analysis. Am J Prev Med.

[B14] Mahmoud MH, Elderiny SN (2018). Effect of lifestyle modification intervention on health status of coronary artery disease patients: Randomized Control Trial. Int J Nurs Stud.

[B15] Moyer VA (2012). Screening for prostate cancer: US Preventive Services Task Force recommendation statement. Ann Intern Med.

[B17] Odedina F, Oluwayemisi AO, Pressey S (2014). Development and assessment of an evidence-based prostate cancer intervention programme for Black men: The WORD on prostate cancer video. Asia Pac J Clin Oncol.

[B18] Polit DF, Beck CT (2010). Essentials of nursing research: Appraising evidence for nursing practice. Lippincott Williams and Wilkins.

[B20] Saleh AM, Fooladi MM, Petro-Nustas W, Dweik G, Abuadas MH (2015). Enhancing knowledge, beliefs, and intention to screen for prostate cancer via different health educational interventions: a literature review. Asian Pac J Cancer Prev.

[B21] Sheehan CA (2009). A brief educational video about prostate cancer screening: a community intervention. Urol Nurs.

[B24] Taylor KL, Davis JL, Turner RO (2006). Educating African American men about the prostate cancer screening dilemma: a randomized intervention. Cancer Epidemiol Biomarkers Prev.

[B25] Taylor KL, Turner RO, Davis III JL (2016). Improving knowledge of the prostate cancer screening dilemma among African American men: an academic-community partnership in Washington, DC. Public Health Rep.

[B26] Torre LA, Bray F, Siegel RL (2015). Global cancer statistics, 2012. CA Cancer J Clin.

[B27] Ukoli FA, Patel K, Hargreaves M (2013). A tailored prostate cancer education intervention for low-income African Americans: Impact on knowledge and screening. J Health Care Poor Underserved.

[B28] Weinrich SP, Seger R, Miller BL (2004). Knowledge of the limitations associated with prostate cancer screening among low-income men. Cancer Nurs.

[B29] Wilkinson S, List M, Sinner M, Dai L, Chodak G (2003). Educating African-American men about prostate cancer: impact on awareness and knowledge. Urology.

[B30] World Health Organization (WHO) (2012) World Health Statistics.

